# Survivors of war in the Northern Kosovo (II): baseline clinical and functional assessment and lasting effects on the health of a vulnerable population

**DOI:** 10.1186/1752-1505-4-16

**Published:** 2010-09-21

**Authors:** Shr-Jie Wang, Sebahate Pacolli, Feride Rushiti, Berina Rexhaj, Jens Modvig

**Affiliations:** 1 Rehabilitation and Research Centre for Torture Victims (RCT), Copenhagen, Denmark; 2 Kosova Rehabilitation Centre for Torture Victims (KRCT), Pristina, Kosovo; 3 Department of Psychology, University of Pristina, Kosovo

## Abstract

**Background:**

This study documents torture and injury experience and investigates emotional well-being of victims of massive violence identified during a household survey in Mitrovicë district in Kosovo. Their physical health indicators such as body mass index (BMI), handgrip strength and standing balance were also measured. A further aim is to suggest approaches for developing and monitoring rehabilitation programmes.

**Methods:**

A detailed assessment was carried out on 63 male and 62 female victims. Interviews and physical examination provided information about traumatic exposure, injuries, and intensity and frequency of pain. Emotional well-being was assessed using the "WHO-5 Well-Being" score. Height, weight, handgrip strength and standing balance performance were measured.

**Results:**

Around 50% of victims had experienced at least two types of torture methods and reported at least two injury locations; 70% had moderate or severe pain and 92% reported constant or periodic pain within the previous two weeks. Only 10% of the victims were in paid employment. Nearly 90% of victims had experienced at least four types of emotional disturbances within the previous two weeks, and many had low scores for emotional well-being. This was found to be associated with severe pain, higher exposure to violence and human rights violations and with a low educational level, unemployment and the absence of political or social involvement.

Over two thirds of victims were overweight or obese. They showed marked decline in handgrip strength and only 19 victims managed to maintain standing balance. Those who were employed or had a higher education level, who did not take anti-depressant or anxiety drugs and had better emotional well-being or no pain complaints showed better handgrip strength and standing balance.

**Conclusions:**

The victims reported a high prevalence of severe pain and emotional disturbance. They showed high BMI and a reduced level of physical fitness. Education, employment, political and social participation were associated with emotional well-being. Interventions to promote physical activity and social participation are recommended. The results indicate that the rapid assessment procedure used here offers an adequate tool for collecting data for the monitoring of health interventions among the most vulnerable groups of a population exposed to violence.

## Background

Ending a war does not put an end to its effects. The long-lasting physical and psychological harm suffered by individuals has been studied in various countries[1-4]. Beyond the physical and psychological consequences associated with torture trauma, ethnic, cultural, social and political contexts influence the coping patterns of individuals[2,5]. A few studies have considered the pre-trauma and post-trauma factors that favour the resumption of normal life for a population in a particular setting[6-8]. The present study, following the household survey which described the prevalence and risk factors of experience of violence and human rights violations in Mitrovicë district[7], examined in detail the experience and the present situation of a group of victims of massive violence. The study enquired into the emotional and physical fitness of a vulnerable population and looked at factors affecting their return to normal life.

Kosovo has suffered from many years of violent confrontation and from tensions between Albanian and Serbian communities. During the Tito era (1945-1986), the Yugoslav government granted Kosovo autonomous status within the republic of Serbia. However, in March 1989, Slobodan Milošević abrogated Kosovo's constitutional autonomy and purged hospitals, schools and civil service of most Albanian workers, which caused very high unemployment in Kosovo and exacerbated the tensions between the ethnic Albanians and the Serbs[9,10]. Political repression and economic deprivation sparked nationalist dissidence and a struggle for self-determination by the Kosovo Liberation Army (KLA). Since 1996, the KLA intensified its attacks on Serbian authority. In response, in March 1998, the Serbian armed forces surrounded the KLA leader and his associates in Donji Prekaz in Skënderaj municipality. A series of armed clashes resulted in mass casualties both among militia and civilians. The incident provoked outrage among ethnic Albanians in Kosovo and also in Western countries. The psychological impact paved the way for a wider resistance movement and hostility between Albanians and Serbs, which has not been overcome.

Several population-based studies have shown the immediate health impact of the Kosovo war on the conflict-affected population[11-14]. The pre-war and post-war experience of ethnic conflict is endogenous, embedded within a complex personal, socio-economic and political matrix. Some victims have resumed a normal life in post-war Kosovo, but others still suffer from both the direct consequences of the war and the associated violence and from long-term effects on their development and well-being. The Kosova Rehabilitation Centre for Torture Victims (KRCT) provided treatment for 1,772 trauma victims across Kosovo from 2004 to 2008 and intends to improve its facility-based service and community health programme. In developing a rehabilitation strategy, it is important to document traumatic experience and to assess its long-lasting effects for the emotional and physical fitness and social functioning of victims of massive violence, and secondly, to look at the factors that help survivors to cope with the trauma. In our household survey in 2008[7], we found that nearly 20% of the population of Mitrovicë district suffered from physical or mental pain. Families affiliated with the Kosovo Liberation Army were especially likely to have been subjected to massive violence and human rights violations before and during the war. However, members of these families were less likely to report pain complaints during the survey. In the present study, a group of victims of massive violence, identified in the household survey, was recruited for a detailed study of their traumatic experience, the effect of different factors on their ability to cope with life and their present health condition. There have been many studies of the effects of the Yugoslav wars and the Kosovo war on mental health, but surprisingly little is known about the nutritional status and physical functioning of victims exposed to massive violence. In this study, we looked at both the emotional and physical fitness of the survivors and examined how various personal factors, inter-personal relationships and the extent of political involvement and social participation interact with emotional and physical fitness. We hypothesised that the victims of massive violence would have poor nutritional status, emotional well-being and performance in physical functioning.

Apart from investigating the present situation, our study was designed to provide information that could help in the development of effective strategies for rehabilitation in this setting. A further aim was to test a simple and rapid tool for assessment of health status of victims of massive violence, which had already been used in a previous study in Bangladesh[5,15]. Such a tool, especially if it can be used in different settings, could provide a useful basis for designing appropriate rehabilitation strategies and also be valuable for monitoring rehabilitation programmes.

## Methods

### Study design and implementation

The study was conducted in three Albanian-dominated areas of Mitrovicë district: Mitrovicë, Skënderaj and Vushtrri municipalities from September to October 2008[7]. The study design was based on a simple and rapid assessment protocol previously developed and tested in a study in Bangladesh. The assessment consists of two components: a household survey and a subsequent detailed examination of a group of victims identified in the household survey. The details are described elsewhere [5,15].

The criteria for inclusion in the detailed study were the following experiences, reported by the families during the household survey: 1) torture and other cruel, inhuman or degrading treatment or punishment (TCIDTP); 2) sexual harassment, molestation, rape or insertion of a blunt object into a genital organ and/or the rectum; 3) arrest and detention without warrant or order; or 4) extrajudicial execution of family members, perpetrated by members of law enforcement agency. The definition of torture strictly followed the United Nations Convention against Torture and Other Cruel, Inhuman or Degrading Treatment or Punishment[16]. Altogether, 383 families with members who fulfilled the criteria were identified and they were invited to attend a mobile clinic for a more detailed physical and functional assessment. The selected families were given vouchers that outlined the objectives and the procedure of the study, including the offer of a free medical examination and treatment in the municipal family health centre. If a valid telephone number was available, the families were also contacted by phone before the deployment of three mobile clinics on 9-11 October, 2008. Transportation from the villages to the mobile clinic was arranged on request. Victims with severe mental illness or mental retardation had to be accompanied by a family member. A total of 126 victims took part in the examinations. The objectives and procedure of the study, guarantees of anonymity and confidentiality and the use of the data were explained when victims first arrived at the mobile clinic.

The mobile clinic team consisted of one coordinator, three medical doctors, one physiotherapist, one clinical psychologist from KRCT and 10 interviewers recruited from the Department of Psychology, University of Pristina. The medical doctors and the clinical psychologist had substantial experience in assessing and helping people with post-traumatic stress disorder (PTSD) and other mental disorders. All the team members attended a training workshop, where the principles of the study instruments and procedures were explained. They took the parts of the interviewer and respondent in a role-play practice and also practised doing interviews and physical measurements with the instructors and few patients.

### Instruments used during the assessment

The trained interviewers used a structured questionnaire to collect personal information and elicit trauma experience. Information on physical functioning and activity, participation in social life and environmental factors was obtained in further interviews using a questionnaire based on the WHO International Classification of Functioning, Disability and Health[5,17]. Subjective difficulties with mobility and body functioning were assessed on the following scale: "no", "yes" and "yes with some difficulty". Baseline pain level was assessed using a 4-point pain frequency and intensity scale. Perceived emotional well-being was assessed by the "WHO-5 Well-Being" questionnaire including five questions. For each, scores are given from 0 to 5. The scores for the five questions were summed to create a raw score from 0 to 25. A raw score of 0 represents the worst possible and the highest raw score of 25 represents the best possible quality of life. A raw score under 13 indicated poor emotional well-being and represented a poor quality of life. All questionnaires were translated into Albanian and Serbian.

The medical doctors carried out routine consultation and examination including a blood pressure measurement. All the injuries were noted using body diagrams to record the location, following the guidelines of the Istanbul Protocol: the UN manual on the effective investigation and documentation of torture and other cruel, inhuman or degrading treatment or punishment[18].

To assess physical fitness, the Body Mass Index (BMI) was calculated and muscle strength and equilibrium were tested. For calculation of the BMI, height and weight measurements were taken in a standing position without shoes. Muscle strength was assessed by measuring handgrip strength using a Jamar^® ^hydraulic hand dynamometer. This is a simple and easily-administered procedure, widely used for the measurement of the loss of hand strength[19-21], for outcome documentation after injuries of upper extremities[22], as a functional index of nutritional status and for determination of impairment[23,24]. Handgrip strength was measured for the left and right hands, according to the recommended procedure of the American Society of Hand Therapists[25]. All participants were included, as none had upper limb deformities. After a demonstration by a trained interviewer or physiotherapist, each participant held the hand dynamometer in a standard position. We collected three measurements for each hand and used the highest value in all the analyses. Lacking the reference value for the general population in Kosovo and former Yugoslavia, we measured the handgrip strength of 72 female and 57 male employees of public and private health facilities (administrators, health and maintenance workers), matched as far as possible to the victims by sex and age, as well as municipality and residence location. The mean values for this group were used as reference values.

The ability to maintain physical equilibrium was assessed by a standard standing balance test. The method has been described in detail elsewhere[5]. If balance on one leg was held for more than 30 seconds, it was considered as a successful outcome[26]. None of the participants was blind or had deformities of lower limbs, so all could be tested.

### Statistical analysis

Data were entered and validated in Microsoft Access 2000. Two times 100% cross-checking and one time 5% cross-checking were performed for quality control. Any discrepancy was eliminated by examining the original paper survey forms. One record was mismatched and consequently eliminated. Data analyses were carried out for 125 victims with Stata software, version 11.0 (StataCorp LP, Texas, USA, 2009). The null hypothesis was rejected at the 5*% *significance level (P < 0.05). We carried out a univariate analysis that included mean, standard deviation (SD) and 95% confidence interval (CIs). Bivariate analyses for differences in means were carried out using a two-sample Kolmogorov-Smirnov test or a generalised linear model adjusted for the effects of other variables and confounding factors. The Shapiro-Wilk test and skewness and kurtosis tests were used to determine the normal distribution and homogeneity of variance of height, weight and handgrip strength. Multiple regression analyses were carried out with handgrip strength as a dependent variable and a set of anthropometric variables as independent variables.

### Ethical evaluation

The Declaration of Helsinki and Danish law were adhered to in the course of the study. Research approval was granted by the Ethics Committee of the Academy of Medical Sciences of Kosovo. All of the study participants gave oral informed consent; the information provided was kept confidential. Brief counselling was offered for simple cases of torture or abuse by the medical doctors and clinical psychologist. Severe cases were referred to the municipal family health centres where a group of health professionals trained by KRCT will follow up the cases.

## Results

### Socio-demographic profile of victims of massive violence

Table 1 shows the characteristics of the group of 125 victims recruited in the study (one case had to be discarded owing to inconsistency). All of them were Albanians. The mean age was 47.7. Approximately 50% were 35-55 years old. Only 10% had jobs: they experienced a similar level of violence exposure and human rights violations to those who were unemployed, retired or doing unpaid household work. When asked about their personal income, 35% (n = 44) of victims reported earning less than 50 € per month and around 45% (n = 56) of victims reported that their personal income in the current year (October 2007-September 2008) was higher than in the year they were attacked or tortured.

**Table 1:** Socio-demographic profile of victims, n = 125

Socio-demographic data	Variables	No. of victims (%)
Mitrovicë district	Mitrovicë municipality	40 (32.0)
	Skënderaj municipality	59 (47.2)
	Vushtrri municipality	26 (20.8)
Marital status	Single	8 (6.5)
	Married	98 (79.7)
	Divorced	17 (13.8)
Religion	None	2 (1.6)
	Muslim	123 (98.4)
Education level	None	18 (14.4)
	Primary	62 (49.6)
	Secondary	34 (27.2)
	College or university	8 (6.4)
	Post-graduate	1 (0.8)
	Other	2 (1.6)
Occupation	Not working	41(32.8)
	Household work	40 (32.0)
	Business	1 (0.8)
	Service, journalist or teacher	11 (8.8)
	Pension	30 (24.0)
	Other	2 (1.6)
Monthly income of individual	0 €	16 (12.8)
	0 < x ≤ 50 €	28 (22.4)
	50 < x ≤ 100 €	55 (44.0)
	100 < x ≤ 200 €	15 (12.0)
	200 < x ≤ 400 €	10 (8.0)
	x > 400 €	1 (0.8)
Involved in political party	No involvement	113 (90.4)
	Democratic League of Kosovo (LDK)	2 (1.6)
	Democratic Party of Kosovo (PDK)	8 (6.4)
	Other political party	1 (0.8)
Level of political affiliation	Supporter	92 (73.6)
	Member	5 (4.0)
	Activist	0 (0)
	Leader	2 (1.6)
Often hold a meeting at home or attend a meeting in the community	No	88 (70.4)
	Yes	37 (29.6)
Have personal, financial or political conflict with people of other ethnicities	No	103 (82.4)
	Yes	22 (17.6)
Have ever participated in a demonstration, a strike or a human rights rally at some time	No	65 (52.0)
	Yes	60 (48.0)
Have family member who worked with Kosovo Liberation Army (KLA) or militia before or during the war in 1999	No	73 (58.4)
	Yes	52 (41.6)
Have relative or friend working with law enforcement agency before or during the war	No	119 (95.2)
	Yes	6 (4.8)
Have relative or friend involved in illegal activity	No	123 (98.4)
	Yes	2 (1.6)

#### *Political activity and social life*

Table 1 shows that 48% of victims had participated in a demonstration, a strike or a human rights rally at some time and 30% said they often attended or held meetings. Approximately 42% reported that their family members worked with the Kosovo Liberation Army or militia before or during the war. Only 8% were involved in a political party.

Over 75% said they had good friends in whom they could confide and who could help them when they had difficulties; 60% have been out to visit their friends during the previous two weeks (which was at the end of month of Ramadan and during the 3-day Eid festival). Although 98% were Muslims, only 27% had participated in any religious or spiritual activities within the seven days preceding the study. Concerning fear of violence in the community, 33% of victims said that they were often afraid or always afraid.

### Trauma experience and present health status

Over half of victims had experienced at least two types of torture methods or reported bodily injury in at least two locations (Tables 2 &3). Men tended to have experienced more torture methods than women. Around 50% of the victims reported forehead and head injury and 40% reported chest injury. When asked about severity of pain experienced during two weeks preceding the survey, 70% reported moderate or severe pain, and when asked about frequency of this pain, 92% reported constant or periodic pain (Table 3). Prevalence of emotional disturbances within the previous two weeks was high: 90% felt angry, 60% felt hate and 80% suffered from sleep disorder. Women reported both crying and feeling sad significantly more often than men (Table 3).

**Table 2:** Injury reporting by the victims, n = 125

Numbers of reported injuries on the body map	No. of victims (%)
0	34 (27.2)
1	29 (23.2)
2	26 (20.8)
3	23 (18.4)
4	8 (6.4)
≥ 5	5 (4.0)
**Body mapping of injury**	**No. of victims (%)**
Forehead	29 (23.2)
Head	34 (27.2)
Neck	17 (13.6)
Shoulder	10 (8.0)
Chest	53 (42.4)
Upper back	19 (15.2)
Arms	14 (11.2)
Lower back and abdomen	7 (5.6)
Legs	21 (16.8)
Ankles	4 (3.2)
Toes	1 (0.8)
Feet soles	3 (2.4)
Fingers of both hands	4 (3.2)
Palms of both hands	6 (4.8)
Genital organ	3 (2.4)

**Table 3:** Experience of torture methods, pain complaints and emotional disturbances reported by the victims, n = 125

Number of torture methods experienced	Male (n)	Female (n)	Total (%)	Difference between male and female by Kolmogorov-Smirnov test corrected P value
0	8	14	22 (17.6)	P < 0.001
1	8	18	26 (20.8)	
2	9	14	23 (18.4)	
3	13	2	15 (12.0)	
4	3	3	6 (4.8)	
≥ 5	22	11	33 (26.4)	
**Pain severity within two weeks**	**Male (n)**	**Female (n)**	**Total (%)**	
No pain	3	4	7 (5.8)	P = 0.925
Light pain	12	17	29 (24.2)	
Moderate pain	28	18	46 (38.3)	
Severe pain	17	21	38 (31.7)	
**Pain frequency within two weeks**	**Male (n)**	**Female (n)**	**Total (%)**	
Constant pain (all the time)	26	23	49 (45.4)	P = 1
Periodic pain (one or more times a week)	24	26	50 (46.3)	
Occasional pain (less than once a week)	5	4	9 (8.3)	
**Emotional disturbance within two weeks**	**Male (n)**	**Female (n)**	**Total (%)**	
Anger	60	54	114 (91.2)	P = 0.977
Aggressiveness	45	36	81 (64.8)	P = 0.558
Crying	35	53	88 (70.4)	P < 0.005
Family non-cooperative	41	41	82 (65.6)	P = 1
A feeling of being insulted	24	35	59 (47.2)	P = 0.187
Hatred	33	43	76 (60.8)	P = 0.263
Helplessness	42	47	89 (71.2)	P = 0.936
Inferiority complex	39	42	81 (64.8)	P = 1
Loss of interest	42	42	84 (67.2)	P = 1
Memory loss	44	46	90 (72.0)	P = 1
Police or military phobia	44	42	86 (68.8)	P = 1
Sadness	27	46	73 (58.4)	P < 0.005
Sexual dysfunction	37	31	68 (54.4)	P = 0.956
Sleep disorder	49	51	100 (80.0)	P = 1
Social isolation	43	41	84 (67.2)	P = 1
Hopelessness	43	42	85 (68.0)	P = 1
**Number of emotional disturbances**	**Male (n)**	**Female (n)**	**Total (%)**	
0	3	2	5 (4.0)	P = 0.951
1 to 3	6	2	8 (6.4)	
4 to 7	5	6	11 (8.8)	
8 to 11	16	16	32 (25.6)	
≥12	33	36	69 (55.2)	

In total, 22 (18%) of the victims were diagnosed with hypertension, 59 (47%) with PTSD, 72 (58%) with depression and 46 (37%) with anxiety disorder, three were mentally retarded, three had phobias, three had psychoses. There was one with bipolar disorder, one who suffered from panic attacks, one with autism and one with neurosis. Sixty-six victims (53%) were currently taking medications against depression or anxiety. People who were ill or took regular medications didn't fast during the month of Ramadan.

#### *Perceived emotional well-being*

Out of the 125 victims who completed the "WHO-5 Well-Being" questionnaire, 96 (77%) scored less than 13, which indicated poor emotional well-being and quality of life. The relationship between perceived emotional well-being and other factors was examined using a generalised linear model (Table 4). Age, sex, number of torture methods experienced and number of injuries or location of bodily injury did not yield a significant effect associated with a poor score. A poor score was associated with various personal factors like unemployment, lower education and income level. Poor emotional well-being was also associated with the following variables related to personal experience: higher exposure to violence and human rights violations, higher pain intensity, experience of at least four types of negative emotional disturbances within the previous two weeks and taking medications against depression or anxiety. In contrast, individuals who have ever taken part in demonstrations, strikes or human rights rallies generally scored well.

**Table 4:** Emotional well-being and its association with personal factors and health condition

Variables (WHO-5 Well-Being < 13, poor emotional well-being)	OR (95% CI)	P value
Political party member vs. general party supporter	0.50 (0.08-3.20)	0.463
Political leader vs. general party supporter	0.33 (0.02-5.5)	0.444
Often attend meeting or hold meeting at home	1.43 (0.55-3.71)	0.464
Have participated in demonstration, a strike or a human rights rally at some time	0.32 (0.13-0.78)	< 0.05
Have conflict with people of other ethnicities	0.58 (0.21-1.60)	0.295
Exposure to 1-3 categories of organised crime or political violence	7.00 (1.17-41.76)	< 0.05
Exposure to at least 4 categories of organised crime or political violence	8.44 (1.33-53.51)	< 0.05
Number of torture methods experienced	1.12 (0.92-3.16)	0.258
Number of bodily injury reported by the victims	1.32 (0.76-2.32)	0.328
Rarely have fear of violence in the community vs. no fear of violence	0.94 (0.26-3.37)	0.92
Sometimes have fear of violence in the community vs. no fear of violence	2.29 (0.70-7.44)	0.17
Often have fear of violence in the community vs. no fear of violence	3.12 (0.77-12.58)	0.11
Always have fear of violence in the community vs. no fear of violence	8.84 (1.06-74.03)	< 0.05
Having 1-3 types of emotional disturbances within 14 days vs. no emotional disturbance	2.40 (0.18-32.88)	0.512
Having at least 4 types of emotional disturbances within 14 days vs. no emotional disturbance	18.40 (1.95-173.53)	< 0.01
Light pain within 14 days vs. no pain	4.09 (0.67-24.83)	0.126
Moderate pain within 14 days vs. no pain	16.67 (26.2-106.08)	< 0.005
Severe pain within 14 days vs. no pain	13.33 (2.08-85.41)	< 0.01
Taking medications against depression or anxiety	2.66 (1.12-6.33)	< 0.05
Income level 0<x≤50 € vs. no income	0.43 (0.08-2.37)	0.332
Income level 50<x≤100 € vs. no income	0.51 (0.10-2.57)	0.416
Income level 100<x<≤200 € vs. no income	0.57 (0.08-4.01)	0.573
Income level 200<x≤400 € vs. no income	0.14 (0.02-0.99)	< 0.05
Income level >400 € vs. no income	0	-
Employment: household work vs. not working	0.37 (0.10-1.32)	0.128
Employment: business vs. not working	0	-
Employment: service, journalist or teacher vs. not working	0.13 (0.27-0.63)	< 0.05
Employment: pension vs. not working	0.25 (0.07-0.92)	< 0.05
Education level	0.65 (0.43-0.98)	< 0.05
Age ≥55 vs. age under 55 year old	1.01 (0.41-2.48)	0.982
Female vs. male	1.54 (0.66-3.57)	0.314

#### *Physical characteristics and measurements of physical functioning*

The values for height, weight and handgrip strength were normally distributed according to the Shapiro-Wilk test and skewness and kurtosis tests. The average weight of male and female victims was 77.8 kg and 71.9 kg, respectively (Table 5). Over two thirds of male and female victims were overweight (25.0 < BMI < 30.0) or obese (BMI > 30). Women tended to have higher BMI than men.

**Table 5:** Health indicators for the group of victims and the group of health facility employees

Health indicators	Victims		Employees at health facilities	
**Age group**	**Male: n (%)**	**Female: n (%)**	**Male: n (%)**	**Female: n (%)**
0-14	3 (4.8)	2 (3.2)	0	0
15-24	3 (4.8)	2 (3.2)	6 (10.5)	3 (4.2)
25-34	8 (12.7)	7 (11.3)	18 (31.6)	16 (22.2)
35-44	15 (23.8)	16 (25.8)	14 (24.6)	10 (13.9)
45-54	16 (25.4)	14 (22.6)	9 (15.8)	31 (43.1)
55-64	7 (11.1)	12 (19.4)	8 (14.0)	12 (16.3)
≥65	11 (17.5)	9 (14.5)	2 (3.5)	0
**Body size**	**Male: mean (95% CI)**	**Female: mean (95% CI)**	**Male: mean (95% CI)**	**Female: mean (95% CI)**
Height (cm)	168.6 (166.8-170.3)	155.3 (152.8-157.7)	175.7 (173.7-177.7)	164.4 (162.8-165.9)
Weight (kg)	77.8 (74.6-80.9)	71.9 (67.2-76.6)	79.7 (76.9-82.6)	70.3 (67.5-73.1)
**Body mass index (BMI: kg/m^2^)**	**Male: n (%)**	**Female: n (%)**	**Male: n (%)**	**Female: n (%)**
BMI<16.5	0	1 (1.6)	0	0
16.5≤BMI<18.5	0	1 (1.6)	0	2 (2.8)
18.5≤BMI<25	18 (28.6)	15 (24.2)	21 (36.8)	28 (38.9)
25≤BMI<30	23 (36.5)	16 (25.8)	30 (56.2)	26 (36.1)
BMI≥30	19 (30.2)	28 (45.2)	6 (10.5)	16 (22.2)
Missing	3 (4.8)	1 (1.6)	0	0
**Hand grip strength**	**Male: mean (95% CI)**	**Female: mean (95% CI)**	**Male: mean (95% CI)**	**Female: mean (95% CI)**
Right hand (kg)	28.5 (25.2-31.7)	22.3 (20.0-24.6)	46.9 (45.1-48.8)	30.7 (29.5-31.9)
Left hand (kg)	27.1 (23.7-30.4)	20.2 (18.0-22.5)	45.0 (42.1-47.9)	27.6 (25.6-29.6)

Only 38 victims (33%) reported that they were able to carry a load of shopping without any difficulty and their self-report of physical functioning and outcome of their handgrip strength measurement was found to be strongly related (coef = 6.36, P < 0.05). The mean handgrip strength of the dominant hand was 30.5 kg (95% CI: 27.3-33.7) for male victims and 23.0 kg (95% CI: 20.6-25.5) for female victims. The mean handgrip strength for the dominant hand for male employees of the health facilities was 48.2 kg (95% CI: 46.2-50.1) and for female employees 31.2 kg (95% CI: 30.0-32.3). The left hand was dominant in 42 out of 125 (37%) victims and in 32 out of 129 (25%) employees of the health facilities. The strength ratio was 1.29 (95% CI: 1.21-1.37) for the victims and 1.71 (95% CI: 1.24-2.19) for the employees at the health facilities, which indicated that the difference in the strength of dominant and non-dominant hands was less in victims. Five victims had no strength in either hand; two had no strength in the right hand, and one had none in the left hand. Only 5 out of 62 male and 10 out of 63 female victims had handgrip strength for the dominant hand equal to or above the mean value of dominant hand for health facility employees of the same sex.

A generalized linear model was used for the following analysis in victims. Handgrip strength was lower in women than in men and declined significantly with increasing age in both sexes. Handgrip strength of dominant hand in victims was not related to BMI, but it was associated with height (coef = 0.30, P < 0.05), weight (coef = 0.13, P = 0.05) and personal factors like education level (coef = 13.68, P < 0.005 for having a college or university degree), as well as income level (coef = 6.82, P < 0.05 for having a monthly income of 200 € or more). A statistically significant decline of handgrip strength performance was observed in individuals with forehead injury (coef = -5.86, P < 0.01). Poor handgrip strength was also associated with an emotional disturbance within the previous two weeks, i.e. feelings of sadness (coef = -7.42, P < 0.001) and helplessness (coef = -4.76, P < 0.05) and with lower scores for the WHO-5 Well-Being questionnaire (coef = 0.47, P < 0.05). The association between the use of antidepressant or anti-anxiety medications and poor handgrip strength performance was of borderline significance (coef = -3.82, P = 0.05) adjusted for interaction between age groups and sex.

There were gender differences in victims in the following results. Women with a job showed better handgrip strength performance (coef = 17.00, P < 0.01) than those without a job, whereas with men there was no difference. Having negative emotional disturbances within the two weeks preceding the survey (coef = -19.7, P < 0.05 for having 1-3 types of emotional disturbances and coef = -16.9, P < 0.05 for having at least four types of emotional disturbances) seemed only to affect the handgrip strength outcome among women. On the other hand, married men showed greater handgrip strength than those who were single (coef = 16.17, P < 0.01), whereas married women did not. The result also suggested that pain complaints within the two weeks preceding the survey (coef = -16.15, P < 0.05 for complaints of moderate pain and coef = -19.10, P < 0.01 for complaints of severe pain) were negatively and significantly correlated with handgrip strength performance among men.

There were 79 victims (63%) who reported decrease in physical activity within the previous two weeks. Only 28 victims (25%) stated that they were able to walk to the other side of their village or community without any difficulty and they seemed to have a better standing balance outcome (coef = 0.16, P = 0.07). The mean duration for standing balance to be maintained on the right foot or the left foot was around 11.6 seconds (min-max: 0-58 seconds for right foot, 0-62 seconds for left foot). Only four victims (3%) were able to stand on either foot for 30 seconds while 15 (12%) could maintain their balance standing on one foot or the other for 30 seconds (Table 6). Since the number of victims able to hold standing balance on either leg for 30 seconds was so small, we defined maintaining standing balance on one leg for 30 seconds as a "successful" outcome variable for analysis. No association was found between balance and sex, height, weight or BMI. Individuals over 55 years old had more difficulty in maintaining standing balance than younger people (OR = 0.10, 95% CI = 0.01-0.77, P < 0.05). Controlling for the confounding factor of age, individuals with complaints of severe pain within the previous two weeks tended to have more difficulty in maintaining standing balance than those who did not have pain (OR = 0.06, 95% CI = 0.00-0.68, P < 0.05). A weak association between taking medication against depression or anxiety and maintaining standing balance was also observed (OR = 0.37, 95%CI = 0.13-1.05, P = 0.06). In contrast, individuals who were employed (OR = 4.76, 95% CI = 1.36-16.60, P < 0.05) and who showed higher handgrip strength (coef = 0.01, P < 0.005) were more likely to maintain standing balance.

**Table 6:** Standing balance test of victims, n = 125

Standing balance mean (seconds)	**Male**: mean (95% CI)	**Feale**: mean (95% CI)	Total mean (95% CI)	Difference between male and female by Kolmogorov-Smirnov test corrected P value
Right leg	11.8 (8.4-15.3)	11.3 (8.2-14.4)	11.6 (9.3-13.9)	P = 1
Left leg	12.3 (8.9-15.8)	10.8 (7.9-13.7)	11.6 (9.3-13.8)	P = 0.968
**Standing balance performance**	**Male (n)**	**Female (n)**	**Total**	
Right leg>30 seconds	7	4	11 (8.8%)	P = 1
Right leg≤30 seconds	56	58	114 (91.2%)	P = 1
Left leg>30 seconds	6	6	12 (9.6%)	P = 1
Left leg≤30 seconds	57	56	113 (90.4%)	P = 1

## Discussion

### Representativity of the study group

Ten years after a war has ended, there will inevitably be difficulties in asking victims to take part in a study, especially when some may have been asked to talk about their traumatic experiences in the past and deep-seated problems in the present. We were not able to follow up the people who did not attend the mobile clinic for the examination, so we cannot know how far our sample was representative of the whole population of victims living in Mitrovicë district. It seems probable that since medical attention and transportation were offered, the study participants were those who were most impaired or suffering. People who had a job and resumed a normal life would be less likely to volunteer to take part. At the other extreme, people who were severely ill or depressed may not have had the energy to become involved. It must also be pointed out that our study participants were still living in Kosovo. Many of the victims of the ethnic conflicts during the rule of Slobodan Milošević have emigrated and settled down in other countries. This type of bias must always be considered in conducting epidemiological studies in post-war settings or places where there have been violent conflicts.

Although our sample may not be representative for the whole population of the region who suffered from the ethnic conflict, they had features in common besides a history of trauma. A large proportion of them complained of pain, suffered from sleep disorders and was taking medications against depression or anxiety. They also tended to have low scores for emotional well-being. Besides the victims' self-reported problems with pain and perceived difficulties with various physical activities, objective measurement of physical functioning also showed that many of the victims had problems that affect their ability to cope with daily life and perhaps make them dependent on other family members for doing household chores and for earning a living[27-29].

### Physical characteristics, health condition and employment

One of the instruments we used was the measurement of handgrip strength. The loss of muscle strength clearly makes it difficult to cope with everyday life, and particularly with jobs requiring manual strength. Some studies that have investigated the complex relationships among depression, pain complaints and disability using physical performance measurements[30,31] have suggested that poor handgrip strength is a valuable indicator of disability. We had previously measured handgrip strength in a study of an oppressed population in Bangladesh[5] and found that the victims showed reduced handgrip strength in their dominant hands. This pattern was also observed in this study. We did not have a precisely-matched control group with which to compare the results, but handgrip strength among the victims was markedly lower than among the employees of the health facilities. Many victims reported difficulty in carrying weights. Poor handgrip strength performance in victims was found to be associated with physical size, pain complaints and poorer emotional well-being, which was also related to the level of violence exposure and to consumption of drugs against depression or anxiety. The results also provided evidence of effects of unemployment and a low education level against handgrip strength in victims.

Measurements of BMI showed that the victims tended to be overweight or obese. The factors affecting BMI are extremely complex. Possible causes include having an unbalanced diet and consumption of drugs against depression and anxiety and these in turn may be due to factors such as a low socio-economic background, unemployment and war exposure. Many of these factors were present in victims. An association between PTSD and obesity and inadequate physical activity has been documented previously in other settings[32-34]. However, it is hard to say, to what extent the problem of obesity among the victims is due to their war experience and current problems and how far it reflects a general trend in the population. There are no population-based statistics on obesity and related illness in Kosovo, but 30% of Kosovo civilians were diagnosed with hypertension (often associated with obesity) in a recent survey[35]. There has been an increase in excessive weight and obesity and obesity-related problems in nearby countries too, including post-war Croatia[36] and Bosnia and Herzegovina[37,38]. A similar trend was observed in Albania and for the rural population in Serbia[39].

A further characteristic of the group of victims in our study was that they tended to be shorter in stature than the employees working at the health facilities. This characteristic may have had an effect on their past experiences and on some of their present problems. Many studies of bullying showed that the masculine norm of physical aggression or dominance was associated with body size and strength as well as with social competence and entitlement[40]. It has even been shown that taller people tend to have better success in the workplace[41,42]. It is possible that taller people were less vulnerable and more capable of defending themselves and surviving the hardships of war and of life in post-war Kosovo because they were more physically fit and socially competent.

One of the most striking problems among the victims was that only 10% were in paid employment. It is clear that, in a country with an unemployment rate of 42% in 2008, the generally poor health of the victims would have made it difficult for them to compete in the job market. More specifically, it is known that obese adults with impaired lower extremity performance are less likely to be hired and they experience decreased quality of life[43,44]. We found that in victims not only was obesity common but also standing balance was poor, which was something we also observed in an oppressed population in Bangladesh[5]. Our results also showed that the victims with a job achieved better outcome in maintaining standing balance. Many victims showed poor standing balance outcomes and reported difficulties in walking in the neighbourhood. The distance that they can walk could be limited and they may move slowly - this could be one of barriers for them entering employment. It is recommended to conduct a comprehensive balance and mobility assessment for those with poor standing balance performance.

We also considered the question of whether there was a relationship between current employment status of victims and their past history of trauma, but we found no evidence that the victims with a job had been exposed to less violence or human rights violations than the others. However, the employed ones demonstrated better test outcomes for emotional well-being and physical functioning than the others. A further study would be needed to examine why this small group had been able to obtain jobs - whether they were more physically fit, had better education, or had a good social network, or simply had a more resilient character.

### Good and bad effects of being in organisations

One of the important aims of our study was to identify factors that help or hinder the reintegration of victims of massive violence and the return to normality. On one hand, active participation in political and social movements increases the exposure to organised crime and political violence under a regime that represses any potential challenge to its power and resources[5,45]. Around 40% of victims in this study reported their affiliation with the Kosovo Liberation Army or militia while 25% of all families in the household survey reported such an affiliation[7]. Associates or family members of Kosovo Liberation Army fighters tended to be targeted by the law enforcement agencies and paramilitaries. On the other hand, political and social involvement may have brought some psychological benefits as has been mentioned in other studies[46,47], particularly among historically deprived citizens. Our study showed that individuals, who had taken part in demonstrations, strikes or human rights rallies against the authorities and who fought for and supported the self-determination of Kosovo, often scored better for emotional well-being. People who play an active role in the community develop a collective identity, and collective response to repression and violence can generate various mechanisms for resistance, survival, healing and restoration at individual and population level, which should never be underestimated[48]. In addition, affiliation with a group could bring concrete benefits like better access to the job market, financial resources or humanitarian aid.

### Usefulness of the study procedure for planning and monitoring interventions

The problems in Kosovo are not over. In recent years, clustering of ethnic groups and recurrence of violence and growing resistance to Kosovo authority in the northern region and Serb enclaves echo the past ethnic struggle. Unresolved ethnic and identity issues in the past always show up in every conflict in the present. Interventions to promote social participation and community coherence can empower the victims and may improve the emotional well-being of a marginalised population[49,50]. For example, the victims in our study did have some outdoor and social activities, but additional physical exercises to improve physical functioning might be instrumental in increasing mobility and in improving emotional well-being, which may in turn promote community reintegration [28,51,52]. Our results also suggested that emotional vulnerability and reduced physical functioning were associated with various personal factors like unemployment and low education level. One goal of rehabilitation should be to address the particular problems that make it difficult for people to find employment.

Feeling entangled with the past may sabotage emotional well-being of war victims and the peace-building in the region in the future. In order to develop rehabilitation programmes, which aim to reduce emotional vulnerability and to improve mental health, we need to understand what factors could empower victims to deal with their struggle in the past and in the present. The survey procedure we used, which had already been developed in Bangladesh[5,15], allowed us to collect detailed information about a group of 125 victims exposed to massive violence. The data now can be used in the planning of programmes for rehabilitation and prevention of violence in this area, to provide baseline data and to monitor the quality and outcome of interventions.

### Limitations and strengths

A strength of the study was that the methods used for physical assessment were simple and inexpensive. The results provided both objective measurements and confirmation of the subjective difficulties in physical functioning reported by the study participants. The oral reports given by participants were also validated by the physical functioning assessment or medical examination that found injury traces on the body.

The problems of selecting a representative group among victims of massive violence living in a disturbed environment have already been discussed. The health-seeking behaviour of the individuals is a major source of selection bias, which limits the extent to which the findings can be generalized. There is, in addition, some risk of memory bias that might have affected the participants' answers to the questionnaire. Many study participants had suffered from memory loss during the two weeks before the study. Some had been diagnosed with PTSD or depression and there is a tendency for these conditions to cause problems with memory and concentration. Interviewer and physician bias also need to be considered. Data analysis would have been strengthened if standardised data for the general population in Kosovo were available. We did have some reference values, for example, for height, weight and handgrip strength from a group of employees of health facilitates, but they were not strictly matched to the group of victims. However, it would have been difficult to find a control group of largely unemployed people with no experience of violence. The timing of the study may have slightly affected the answers to the questions on actively visiting friends and relatives, because it was conducted shortly after the end of Ramadan, when family gatherings are frequent.

## Conclusions

We have confirmed that a rapid assessment protocol, originally developed in another study in Bangladesh[5,15], to study collective exposure to violence and human rights violation and its health effect, was applicable in northern Kosovo. Many victims in our study had a high prevalence of severe pain and emotional disturbance and a reduced level of physical fitness, which was also related to unemployment and low education level. There is little that a classical rehabilitation programme can do about the poverty and unemployment that are part of the wider socio-economic setting. However, we suggest that a community-based rehabilitation programme with a focus on increasing in physical fitness and mobility and promotion of social participation could help to improve emotional health and reduce the tendency to obesity and the prevalence of emotional disturbances among the most traumatized population in northern Kosovo.

## List of abbreviations

BMI: Body Mass Index; CI: confidence interval; KRCT: Kosovo Rehabilitation Centre for Torture Victims; OR: odds ratio; NATO: North Atlantic Treaty Organization; NGO: non-governmental organisation; PTSD: post-traumatic stress disorder; RCT: Rehabilitation and Research Centre for Torture Victims; SD: standard deviation; TCIDTP: torture and other cruel, inhuman or degrading treatment or punishment; WHO: World Health Organization.

## Competing interests

The authors declare that they have no competing interests. The sponsor had no role in the study design, data collection and analysis. There is no relationship between authors and sponsors, which could potentially bias the results.

## Authors' contributions

SW participated in the design of the study, conducted the field work, analysed and interpreted data and drafted the manuscript. SP, FR and BR assisted in data collection, coordination and supervision. JMO participated in the conception of the work, helped to draft the manuscript and revised it critically at all stages. All the authors read and approved the final manuscript.

## References

[B1] Murthy RS Lakshminarayana R Mental health consequences of war: a brief review of research findings World Psychiatry 2006 5 1 25 30 16757987 PMC1472271

[B2] Alexander A Blake S Bernstein MA The staying power of pain. A comparison of torture survivors from Bosnia and Colombia and their rates of anxiety, depression and PTSD Torture 2007 17 1 1 10 17456901

[B3] Kashdan TB Morina N Priebe S Post-traumatic stress disorder, social anxiety disorder, and depression in survivors of the Kosovo War: experiential avoidance as a contributor to distress and quality of life J Anxiety Disord 2009 23 2 185 196 10.1016/j.janxdis.2008.06.006 18676121 PMC2667796

[B4] Morina N Ford JD Complex sequelae of psychological trauma among Kosovar civilian war victims Int J Soc Psychiatry 2008 54 5 425 436 10.1177/0020764008090505 18786904

[B5] Wang SJ Haque MA Masum SU Biswas S Modvig J Household exposure to violence and human rights violations in western Bangladesh (II): history of torture and other traumatic experience of violence and functional assessment of victims BMC Int Health Hum Rights 2009 9 31 10.1186/1472-698X-9-31 19943932 PMC2796639

[B6] Astin MC Lawrence KJ Foy DW Posttraumatic stress disorder among battered women: risk and resiliency factors Violence Vict 1993 8 1 17 28 8292561

[B7] Wang SJ Salihu M Rushiti F Bala L Modvig J Survivors of the war in the Northern Kosovo: violence exposure, risk factors and public health effects of an ethnic conflict Confl Health 2010 4 11 10.1186/1752-1505-4-11 20509915 PMC2893511

[B8] Khamis V Post-traumatic stress and psychiatric disorders in Palestinian adolescents following intifada-related injuries Soc Sci Med 2008 67 8 1199 1207 10.1016/j.socscimed.2008.06.013 18657343

[B9] Murati V Ahmeti B Kllokoqi S Konjufca G Actors and processes of ethno-national mobilization in Kosovo Human and minority rights in the Life cycle of ethnic conflicts 2007 Pristina: European Academy of Bozen

[B10] Department of State Kosovo Chronology. Timeline of events 1989-1999 relating to the crisis in Kosovo 1999 Washington, DC

[B11] Iacopino V Frank MW Bauer HM Keller AS Fink SL Ford D Pallin DJ Waldman R A population-based assessment of human rights abuses committed against ethnic Albanian refugees from Kosovo Am J Public Health 2001 91 12 2013 2018 10.2105/AJPH.91.12.2013 11726386 PMC1446925

[B12] Lopes Cardozo B Vergara A Agani F Gotway CA Mental health, social functioning, and attitudes of Kosovar Albanians following the war in Kosovo Jama 2000 284 5 569 577 10.1001/jama.284.5.569 10918702

[B13] American Association for the Advancement of Science (AAAS) Science and Human Rights Program, American Bar Association (ABA) Central and East European Law Initiative (Eds.) Political Killings in Kosova/Kosovo, March-June 1999: A cooperative report by the Central and East European law initiative of the American Bar Association and the Science and Human Rights Program of the American Association for the Advancement of Science 2000 Washington DC: ABA Central and East European Law Initiative

[B14] Spiegel PB Salama P War and mortality in Kosovo, 1998-99: an epidemiological testimony Lancet 2000 355 9222 2204 2209 10.1016/S0140-6736(00)02404-1 10881894

[B15] Wang SJ Modvig J Montgomery E Household exposure to violence and human rights violations in western Bangladesh (I): prevalence, risk factors and consequences BMC Int Health Hum Rights 2009 9 29 10.1186/1472-698X-9-29 19930589 PMC2790439

[B16] United Nations Optional Protocol to the Convention against Torture and other Cruel, Inhuman or Degrading Treatment or Punishment 2006 The Office of the High Commissioner for Human Rights S: United Nations

[B17] International Classification of Functioning, Disability and Health (ICF) http://www.who.int/classifications/icfbrowser/

[B18] United Nations Istanbul Protocol: manual on the effective investigation and documentation of torture and other cruel, inhuman or degrading treatment or punishment 2004 The Office of the High Commissioner for Human Rights, United Nations, Switzerland

[B19] Alperovitch-Najenson D Carmeli E Coleman R Ring H Handgrip strength as a diagnostic tool in work-related upper extremity musculoskeletal disorders in women ScientificWorldJournal 2004 4 111 117 15010565 10.1100/tsw.2004.12 PMC5956401

[B20] Mathiowetz V Kashman N Volland G Weber K Dowe M Rogers S Grip and pinch strength: normative data for adults Arch Phys Med Rehabil 1985 66 2 69 74 3970660

[B21] Mathiowetz V Wiemer DM Federman SM Grip and pinch strength: norms for 6- to 19-year-olds Am J Occup Ther 1986 40 10 705 711 3777107 10.5014/ajot.40.10.705

[B22] Chau N Petry D Bourgkard E Huguenin P Remy E Andre JM Comparison between estimates of hand volume and hand strengths with sex and age with and without anthropometric data in healthy working people Eur J Epidemiol 1997 13 3 309 316 10.1023/A:1007308719731 9258530

[B23] Gunther CM Burger A Rickert M Crispin A Schulz CU Grip strength in healthy Caucasian adults: reference values J Hand Surg [Am] 2008 33 4 558 565 10.1016/j.jhsa.2008.01.008 18406961

[B24] Rantanen T Guralnik JM Foley D Masaki K Leveille S Curb JD White L Midlife hand grip strength as a predictor of old age disability Jama 1999 281 6 558 560 10.1001/jama.281.6.558 10022113

[B25] Fess EE Clinical Assessment Recommendations (2nd edition) 1992 2 Chicago: American Society of Hand Therapists

[B26] Hermodsson Y Ekdahl C Persson BM Roxendal G Standing balance in trans-tibial amputees following vascular disease or trauma: a comparative study with healthy subjects Prosthet Orthot Int 1994 18 3 150 158 7724348 10.3109/03093649409164400

[B27] Hatch J Gill-Body KM Portney LG Determinants of balance confidence in community-dwelling elderly people Phys Ther 2003 83 12 1072 1079 14640866

[B28] Pang MY Eng JJ Miller WC Determinants of satisfaction with community reintegration in older adults with chronic stroke: role of balance self-efficacy Phys Ther 2007 87 3 282 291 10.2522/ptj.20060142 17284545

[B29] Khaodhiar L McCowen KC Blackburn GL Obesity and its comorbid conditions Clin Cornerstone 1999 2 3 17 31 10.1016/S1098-3597(99)90002-9 10696282

[B30] Yanagita M Willcox BJ Masaki KH Chen R He Q Rodriguez BL Ueshima H Curb JD Disability and depression: investigating a complex relation using physical performance measures Am J Geriatr Psychiatry 2006 14 12 1060 1068 10.1097/01.JGP.0000224364.70515.12 17138811

[B31] Panton LB Kingsley JD Toole T Cress ME Abboud G Sirithienthad P Mathis R McMillan V A comparison of physical functional performance and strength in women with fibromyalgia, age- and weight-matched controls, and older women who are healthy Phys Ther 2006 86 11 1479 1488 10.2522/ptj.20050320 17079747

[B32] Llabre MM Hadi F War-related exposure and psychological distress as predictors of health and sleep: a longitudinal study of Kuwaiti children Psychosom Med 2009 71 7 776 783 10.1097/PSY.0b013e3181ae6aee 19592513

[B33] Vieweg WV Fernandez A Julius DA Satterwhite L Benesek J Feuer SJ Oldham R Pandurangi AK Body mass index relates to males with posttraumatic stress disorder J Natl Med Assoc 2006 98 4 580 586 16623072 PMC2569214

[B34] Trief PM Ouimette P Wade M Shanahan P Weinstock RS Post-traumatic stress disorder and diabetes: co-morbidity and outcomes in a male veterans sample J Behav Med 2006 29 5 411 418 10.1007/s10865-006-9067-2 16865552

[B35] Markoglou N Hatzitolios AI Savopoulos Ch G Ziakas AG Koutsopoulos D Metallidis S Epidemiologic characteristics of hypertension in the civilians of Kosovo after the war Cent Eur J Public Health 2005 13 2 61 65 15969452

[B36] Uhernik AI Milanovic SM Anthropometric indices of obesity and hypertension in different age and gender groups of Croatian population Coll Antropol 2009 33 Suppl 1 75 80 19563150

[B37] Pilav A Nissinen A Haukkala A Niksic D Laatikainen T Cardiovascular risk factors in the Federation of Bosnia and Herzegovina Eur J Public Health 2007 17 1 75 79 10.1093/eurpub/ckl066 16698884

[B38] Zec S Telebak B Sljepcevic O Filipovic-Hadziomeragic A Nutrition in pre-war Sarajevo Eur J Clin Nutr 1995 49 Suppl 2 S6 10 8846768

[B39] Pavlica T Bozic-Krstic V Rakic R Body mass index, waist-to-hip ratio and waist/height in adult population from Backa and Banat - the Republic of Serbia Ann Hum Biol 10.3109/03014460903512829 20141483

[B40] Archer J Thanzami V The relation between mate value, entitlement, physical aggression, size and strength among a sample of young Indian men Evolution and Human Behavior 2009 30 5 215 320 10.1016/j.evolhumbehav.2009.03.003

[B41] Judge TA Cable DM The effect of physical height on workplace success and income: preliminary test of a theoretical model J Appl Psychol 2004 89 3 428 441 10.1037/0021-9010.89.3.428 15161403

[B42] Kortt M Leigh A Does Size Matter in Australia?* The Economic Record 2010 86 272 71 83 10.1111/j.1475-4932.2009.00566.x

[B43] Fjeldstad C Fjeldstad AS Acree LS Nickel KJ Gardner AW The influence of obesity on falls and quality of life Dyn Med 2008 7 4 10.1186/1476-5918-7-4 18304350 PMC2288598

[B44] Brandt KD Heilman DK Slemenda C Katz BP Mazzuca S Braunstein EM Byrd D A comparison of lower extremity muscle strength, obesity, and depression scores in elderly subjects with knee pain with and without radiographic evidence of knee osteoarthritis J Rheumatol 2000 27 8 1937 1946 10955336

[B45] Mian A Mahmood SF Chotani H Luby S Vulnerability to homicide in Karachi: political activity as a risk factor Int J Epidemiol 2002 31 3 581 585 10.1093/ije/31.3.581 12055158

[B46] Cicognani E Zani B Dolejšiová D, e García López MA Participation of young people in civil life: the role of sense of community Challenges for citizenship, citizenship education and democratic practice in Europe 2009 Strasbourg Cedex Council of Europe Publishing

[B47] Sanders LM The psychological benefit of political participation Annual Meeting of the American Political Science Association: 2001 2001; San Francisco 2001

[B48] Pedersen D Political violence, ethnic conflict, and contemporary wars: broad implications for health and social well-being Soc Sci Med 2002 55 2 175 190 10.1016/S0277-9536(01)00261-1 12144134

[B49] Cattell V Poor people, poor places, and poor health: the mediating role of social networks and social capital Soc Sci Med 2001 52 10 1501 1516 10.1016/S0277-9536(00)00259-8 11314847

[B50] Weare K Gray G What works in developing children's emotional and social competence and well-being? 2003 Southampton, UK: The Health Education Unit, Research and Graduate School of Education, University of Southampton 115

[B51] Shimada H Uchiyama Y Kakurai S [Relationship between lifestyle activities and physical functions in elderly persons utilizing care facilities] Nippon Ronen Igakkai Zasshi 2002 39 2 197 203 11974945 10.3143/geriatrics.39.197

[B52] Barr EL Browning C Lord SR Menz HB Kendig H Foot and leg problems are important determinants of functional status in community dwelling older people Disabil Rehabil 2005 27 16 917 923 10.1080/09638280500030506 16096244

